# Amniotic fluid-derived exosomes attenuated fibrotic changes in POI rats through modulation of the TGF-β/Smads signaling pathway

**DOI:** 10.1186/s13048-023-01214-1

**Published:** 2023-06-27

**Authors:** Nahideh Nazdikbin Yamchi, Shahin Ahmadian, Halimeh Mobarak, Farhad Amjadi, Rahim Beheshti, Amin Tamadon, Reza Rahbarghazi, Mahdi Mahdipour

**Affiliations:** 1 Stem Cell Research Center, Tabriz University of Medical Sciences, Tabriz, Iran; 2 Faculty of Veterinary Medicine, Shabestar Islamic Azad University, Shabestar, Iran; 3 PerciaVista R&D Co., Shiraz, Iran; 4 Department for Scientific Work, Marat Ospanov Medical University, West, Aktobe, Kazakhstan; 5 Drug Applied Research Center, Tabriz University of Medical Sciences, Tabriz, Iran; 6 Department of Applied Cell Sciences, Faculty of Advanced Medical Sciences, Tabriz University of Medical Sciences, Tabriz, Iran; 7 Department of Reproductive Biology, Faculty of Advanced Medical Sciences, Tabriz University of Medical Sciences, Tabriz, Iran

**Keywords:** Premature ovarian insufficiency, Amniotic derived exosome, Ovarian tissue regeneration, Fertility restoration

## Abstract

In the current study, we investigated the regenerative effects of amniotic fluid exosomes (AF-Exos) in a rat model for premature ovarian insufficiency (POI). POI is a condition characterized by a decrease in ovarian function that can lead to infertility. We induced POI by administering cyclophosphamide (CTX) for 15 consecutive days, and then transplanted AF-Exos directly into both ovarian tissues. Four weeks later, we measured the serum levels of follicle-stimulating hormone (FSH), luteinizing hormone (LH), and estradiol (E2), and performed histopathological evaluations using H & E and Masson’s trichrome staining. We also monitored the expression of genes related to the TGF-β signaling pathway using real-time PCR and examined the fertility rate of POI rats after AF-Exos therapy. Histological analysis showed an increase in atretic follicles and a decrease in healthy follicle count after POI induction. Four weeks post-AF-Exos intervention, the healthy follicle count increased (p < 0.01) while the atretic follicle count decreased (p < 0.001). In parallel, the deposition of collagen fibers also decreased following AF-Exos transplantation. The concentrations of FSH and LH hormones in sera remained unchanged after injection of AF-Exos, while E2 levels increased (p < 0.05). The expression of Smad-4 (p < 0.01) and Smad-6 (p < 0.05) was upregulated in POI rats that received AF-Exos, while Smad-2, TGF-β1, TNF-α, and IL-10 remained statistically unchanged. Our records showed a notable increase in litter number after AF-Exos compared to the non-treated POI rats. These results suggest that AF-Exos transplantation has the potential to restore ovarian function through the TGF-β/Smads signaling pathway in POI rats.

## Introduction

Premature ovarian insufficiency (POI), also known as premature ovarian failure (POF), is characterized by abnormal ovarian function in females under the age of 40 [1]. POI is marked by elevated levels of follicle-stimulating hormone (> 40 IU/mL), decreased levels of estradiol (< 30 pg/mL), and anti-Müllerian hormone (< 1 ng/mL) [2]. The prevalence of POI cases varies between 0.9% and 1.2% in different societies [3]. Several factors such as physiological conditions, genetic traits, autoimmune diseases, infections, surgical procedures, environmental effects, and idiopathic reasons have been linked to POI occurrence [4].

Histological and molecular studies have indicated that POI is characterized by fibrotic changes in ovarian tissue via the engagement of several signaling pathways [5, 6]. Among these, the TGF-β/Smads signaling axis regulates the growth of ovarian follicles at different developmental stages. Dysregulated TGF-β/Smads signaling pathway accounts for follicular atresia and inhibition of follicles, increasing the possibility of POI conditions [7, 8]. Local production of important inflammatory cytokines such as TNF-α and IL-10 has been shown to promote POI consequences [9, 10]. For example, TNF-α induces primary follicle apoptosis, vascular endothelial damage, and reduction of sex-related hormones [7].

Hormone replacement therapy (HRT) is the most common treatment for POI patients, but it lacks complete recovery of ovarian tissue activity and accounts for the occurrence of some female-related malignancies [11–13]. Oocyte and embryo cryopreservation and donation [14], as well as gonadotropin-releasing hormone (GnRH) therapy [15], have also been suggested for POI patients. Unfortunately, these modalities cannot restore normal ovarian tissue activity. Recently, new therapeutic strategies based on stem cells [16–18], and their secretome have been proposed as an effective treatment option for various complications such as infertility [19]. Numerous studies have shown that different cell types can produce and release nano-sized vesicles such as exosomes (Exos), microvesicles, and apoptotic bodies into the extracellular vesicles [20, 21]. Exos, with an average size of 40 to 150 nm, are abundant in several biofluids such as plasma, urine, and amniotic fluid [22, 23]. Exos harbor several signaling molecules that act in a paracrine manner to target cells, resulting in the regulation of specific molecular cascades [24–26]. Amniotic fluid (AF) is an easily accessible biological fluid with significant levels of Exos [27, 28]. AF-derived Exos (AF-Exos) act as mediators of paracrine communication within the intrauterine environment through the transfer of metabolic substances, including proteins, ions, carbohydrates, lipids, enzymes, and hormones, as well as genomic content (dsDNA, ssDNA, miRNA, mRNA), growth factors, and cytokines that support the growth of the placenta and fetus during pregnancy [29–33]. Emerging data have pointed to the fact that Exo-based treatments yield promising therapeutic outcomes for infertility, cancers, infectious diseases, on-target drug delivery, etc. [33–37]. Unlike whole-cell-based therapy, the application of Exos has several advantages such as low immunogenicity, non-toxicity, and ease of access to target cells [38–41]. In the current investigation, we studied the regenerative potential of AF-Exos in a rat model of POI concerning fibrotic changes and the TGF-β/Smads signaling.

## Materials and methods

### Animal issues

Female Wistar rats (n = 27; 7-8-week-old) weighing between 150 and 180 g were purchased from Tehran Medzist Company and maintained at the animal center of Tabriz University of Medical Sciences. All the experiments and interventions performed in this study were approved by the Animal Care and Ethics Committee (IR.TBZMED.VCR.REC.1398.060) and the Guide for the Care and Use of Laboratory Animals (NIH, 1986). Rats were kept in a normal environment with a temperature of 22 ± 2°C, a 12-hour light/dark cycle, and free access to standard chewing pellets and tap water.

### Induction of POI rats

**Fig. 1:**
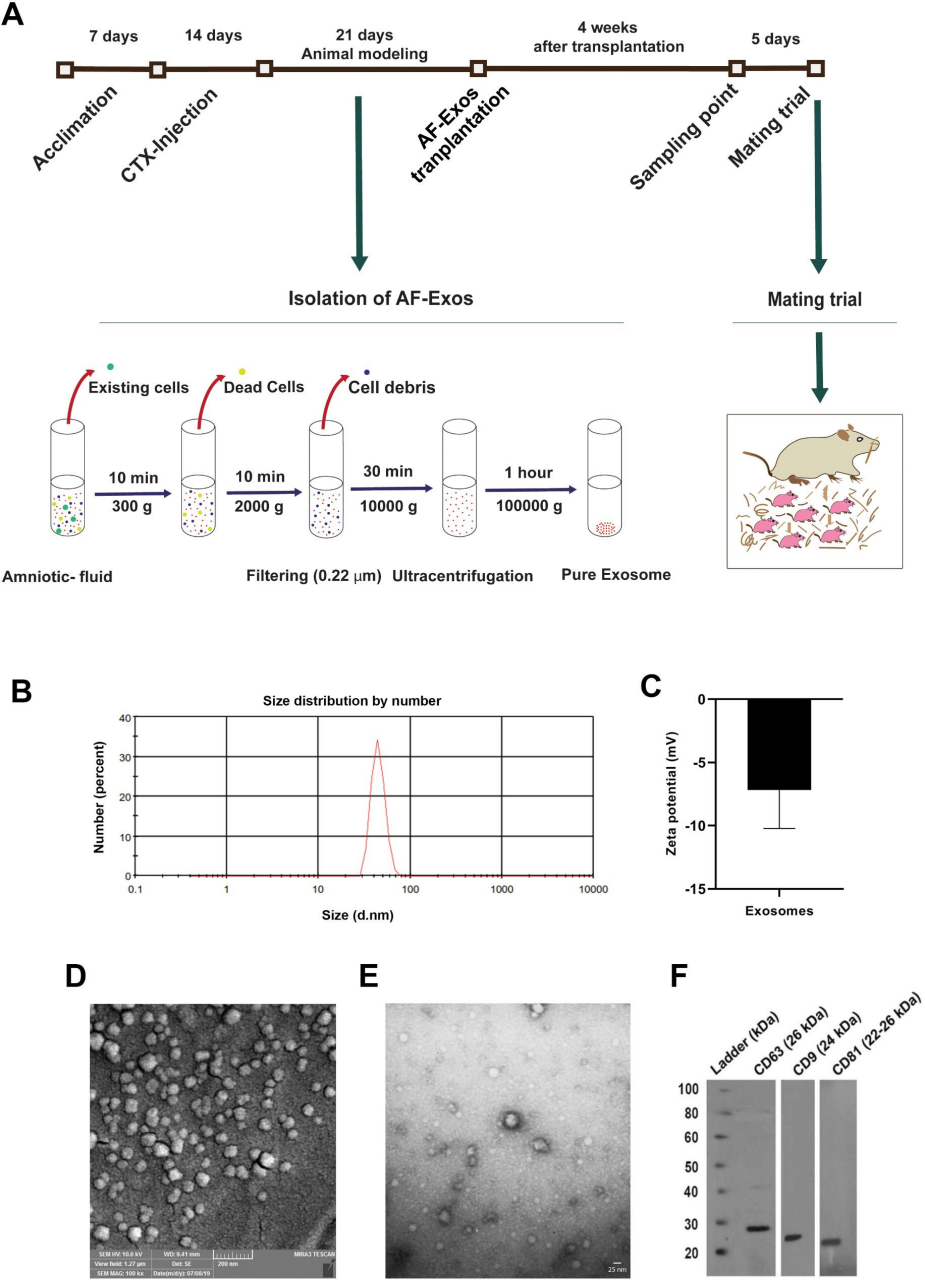
Schematic presentation experimental setup. Animal modeling procedure, exosome enrichment, intervention, sampling and mating **(A)**, characterization of exosomes, size distribution **(B)** and zeta potential **(C)** assessment by dynamic light scattering method, scanning electron microscopy **(D)**, transmission electron microscopy **(E)** and western blot analysis **(F)**

Seven days after acclimation, a POI rat model was induced by the administration of cyclophosphamide (CTX; Cat no: RHRI404, Supelco). In short, CTX was injected intraperitoneally (i.p.) at a dose of 200 mg/kg BW on day 1 and 8 mg/kg bw from days 2 to 14. To confirm the POI status, three rats from the CTX-injected and control groups were randomly selected and subjected to histopathological examination and hormonal evaluations. The remaining POI rats were randomly allocated into three groups as follows: Control (Control-matched POI group, n = 7), Sham (POI + normal saline, n = 7), and AF-Exos (POI + AF-Exos, n = 7) (Fig. 1A).

### Isolation of AF-Exos

AF was obtained from a 3–4 months pregnant ewe without concomitant diseases and metabolic disorders at Tabriz local slaughterhouse under sterile conditions. AF-Exos were enriched by the ultracentrifugation method. To eliminate cells, samples were centrifuged at 300 g for 10 min, and the supernatant was then centrifuged at 2000 g for 15 min to remove dead cells and debris. The procedure was continued by centrifugation of samples at 10,000 g for 30 min. After passing through 0.22-micrometer microfilters, the collected supernatant was subjected to ultracentrifugation for 1 h at a speed of 100,000 g. To remove protein contaminants, a second round of ultracentrifugation was performed, and the pellet was resuspended in phosphate-buffered saline (PBS) and stored at ‒80 °C for subsequent analyses (Fig. 1A) [42, 43].

### AF-Exos characterization

#### Dynamic light scattering (DLS)

To determine size distribution and zeta potential, the DLS approach was performed using a Zetasizer Nanoseries device (Malvern Nano ZS, Herrenberg, Germany), according to the manufacturer’s instructions [44].

### Scanning electron microscopy (SEM)

Samples were fixed with 200 µL pre-cold paraformaldehyde solution (2.5% w/v) (Sigma-Aldrich GmbH, Germany) and lyophilized after ultracentrifugation. Subsequently, samples were gold-sputtered and visualized under a Mira-3 FEG SEM microscope (Tescan, Czech Republic) [45].

### Transmission electron microscopy (TEM)

To further examine the morphology of the isolated AF-Exos, a drop of the exosomal suspension was placed on a 300-mesh copper grid, stained with 2% (w/v) uranyl acetate solution for 15 min, and subsequently covered with a carbon film to prevent degradation under the electron beam. The samples were observed using a TEM instrument (LEO 906, Zeiss, Germany) with a voltage of 100 kV [46].

### Western blotting

Protein levels of exosomal markers were monitored using western blotting, according to our previously published paper [41].

### Ovarian transplantation of AF-Exos

Twenty-one days after the last CTX injection, rats were deeply anesthetized using 90 mg/kg ketamine and 10 mg/kg xylazine. A one-centimeter incision was made in the supra flank region, and ovarian masses were exposed. Using a 25-G syringe, 10 µl of enriched AF-Exos (equal to 10 µg exosomal protein) was injected directly into the ovarian tissue. In the sham group, a similar volume of normal saline was administered.

### Histological examination

Rats were euthanized using an overdose of ketamine and xylazine four weeks after AF-Exos injection (n = 4 per group). Left ovaries were sampled, fixed in 4% formaldehyde, and subsequently embedded in paraffin. Histological sections, with a thickness of 5 μm, were prepared using a microtome instrument. After dehydration in an alcohol series, sections were stained with Hematoxylin and Eosin (H&E) staining solution and evaluated under an Olympus BX51 light microscope. Parameters such as follicle number and quality at four stages of development (primary, secondary, and antral follicles), as well as the existence of corpus luteum (CL), were assessed [47]. To monitor fibrotic changes and collagen fiber deposition, Masson’s trichrome staining was performed [48].

### ELISA analysis

Enzyme-linked immunosorbent assay (ELISA) was performed according to the kit manufacturer’s instructions to measure serum levels of FSH (Cat no: 334-096-4, Monobind), LH (Cat no: 0234 − 96, Monobind), and E2 (Cat no: 4925-300 A, Monobind). Blood samples were obtained from rats under deep anesthesia (90 mg/kg ketamine plus 10 mg/kg xylazine) directly from the heart (n = 4 per group). The samples were then centrifuged at 400 g for 10 min to harvest serum. The collected sera were transferred to cryovials and kept at -80 °C until use.

### Real-time PCR analysis

The fibrosis rate was also assessed four weeks post-intervention by monitoring the expression of Smad-2, -4, -6, Tgf-β1, Tnf-α, and IL-10. To this end, the right ovaries were subjected to RNA extraction using TRIzol reagent (Cat No: 0000124; MaxZol) (n = 4 per group). After determining the quality and concentrations of RNA (NanoDrop Spectrophotometer), cDNA was synthesized using a cDNA synthesis kit (Cat no: YT4500, Yekta Tajhiz Azma). Specific primer pairs were designed using the Primer-Blast online software (https://www.ncbi.nlm.nih.gov/tools/primer-blast/) (Table 1), and specific annealing temperatures were optimized using gradient PCR. Quantitative real-time PCR reactions (qRT-PCR) were set up using SYBR Green 2X (Cat no: 5,000,850, Ampliqon) and cDNA using the Roche Light Cycler 96 system. The PCR reaction consisted of 45 cycles with three stages of denaturation, annealing, and extension at temperatures of (95, 60, and 72 °C, respectively). The specificity of reactions was monitored using melting and melting curves. Related quantifications were elaborated using 2^−ΔΔct^, with β-actin as a housekeeping gene.

**Table 1:** Primer pairs used for gene expression studies

Genes	Sequence (5′➔3′)	Annealing temperature (°C)	Product size (bp)
Rn-β-actin NCBI accession number NM_031144.3	F: TGACAGGATGCAGAAGGAGA	60	104
	R: TAGAGCCACCAATCCACACA		
Rn-TNF-α NCBI accession number NM_012675.3	F: ATGGGCTCCCTCTCATCAGT	60	106
	R: GCTTGGTGGTTTGCTACGAC		
Rn-IL10 NCBI accession Number NM_012854	F: AGTGGAGCAGGTGAAGAATG	60	139
	R: TAGATGCCGGGTGGTTCAAT		
Rn-Smad2 NCBI accession number NM_001277450	F: TCCATCGAACTCGGAGAGGT	60	106
	R: ATACAAGCGCACTCCCCTTC		
Rn-Smad4 NCBI accession number NM_019275.3	F: GCAACCCCCATCACCTTAGT	60	134
	R: CATCGGAGGAAGGTACAGCG		
Rn-Smad6 NCBI accession number NM_001109002.2	F: CGCCTCTATGCGGTGTATGA	60	146
	R: AGCAGGATGCCAAAACCGAT		
Rn-TGF-β1 NCBI accession number NM_021578.2	F: TCCATGACATGAACCGACCC	60	142
	R: TGCCGTACACAGCAGTTCTT		

Abbreviations: NCBI, National Center for Biotechnology Information. F = Forward primer and R = Reverse primer sequences

### Mating trial

To assess the fertility status of the rats, the remaining three rats per group were kept in separate cages with a 2:1 (male: female) ratio for a maximum of one week. After confirming successful mating by observing the vaginal plug, rats were placed in separate cages for three weeks. Finally, the number of offspring was recorded.

### Statistical analysis

All mean ± SEM results were analyzed using GraphPad Prism 8.0.1 software. Data were analyzed using One-way analysis of variance with a post hoc Tukey test. A t-test was also used to find statistically significant differences between the two groups. Values below 0.05 were considered statistically significant.

## Results

### Induction of POI conditions

**Fig. 2:**
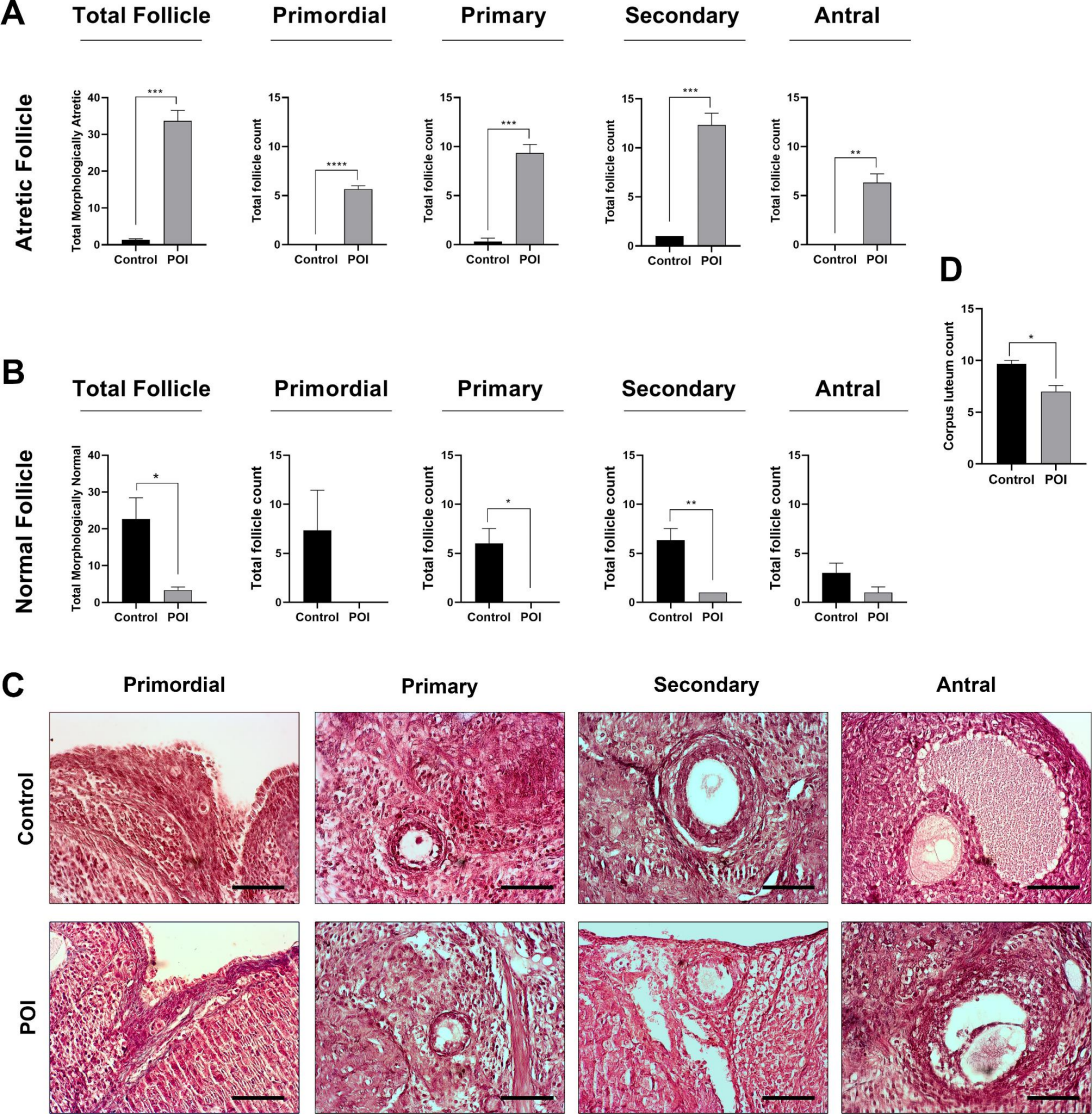
Morphological assessment of ovarian tissue after animal modeling, total atretic and atretic follicles count at different developmental stages of primordial, primary, secondary and antral **(A)**, total healthy and healthy follicles count at different developmental stages of primordial, primary, secondary and antral **(B)**, H&E staining **(C)** and total corpus luteum count **(D)**. (Scale bar = 200 µm), *p < 0.05; **p < 0.01; ***p < 0.001 and ****p < 0.0001 (n = 4)

To confirm the POI condition, a histological evaluation was performed twenty-one days after the last dose of CTX injection, and follicular status was observed within the ovarian tissues (Fig. 2A-D). Bright-field imaging revealed a significant general follicular atresia with cumulus cell disintegration, vacuolation, and separation of granulosa cells recorded at all stages of development in the CTX-received rats compared to the control group (p < 0.05; Fig. 2A-D). On the contrary, a decline pattern was observed in the number of morphologically healthy follicles at all stages of development in the CTX-injected rats compared to the control group (p < 0.05; Fig. 2B). The data also revealed a significant reduction in the number of corpus luteum (CL) in POI rats compared to the non-treated control group (p < 0.05; Fig. 2D). These features confirmed that CTX administration led to POI conditions in rats.

### AF-Exos characterization

We are currently performing several projects related to the application of AF-Exos for the restoration of injured ovarian tissue function. To this end, data from our previously published articles were presented here with permission to reproduce [41]. According to DLS data, the isolated AF-Exos exhibited an average zeta potential of − 7.16 mV and a mean diameter of 50 ± 7.521 nm (Fig. 1B-C). SEM and TEM images indicated typical round and cup-shaped particles, which are identical to Exos (Fig. 1D-E). The isolated Exos harbored specific tetraspanins such as CD63, CD9, and CD81 (Fig. 1F). These features indicated the efficiency of the current protocol to enrich the AF-Exos.

### AF-Exos transplantation and ovarian follicles

**Fig. 3:**
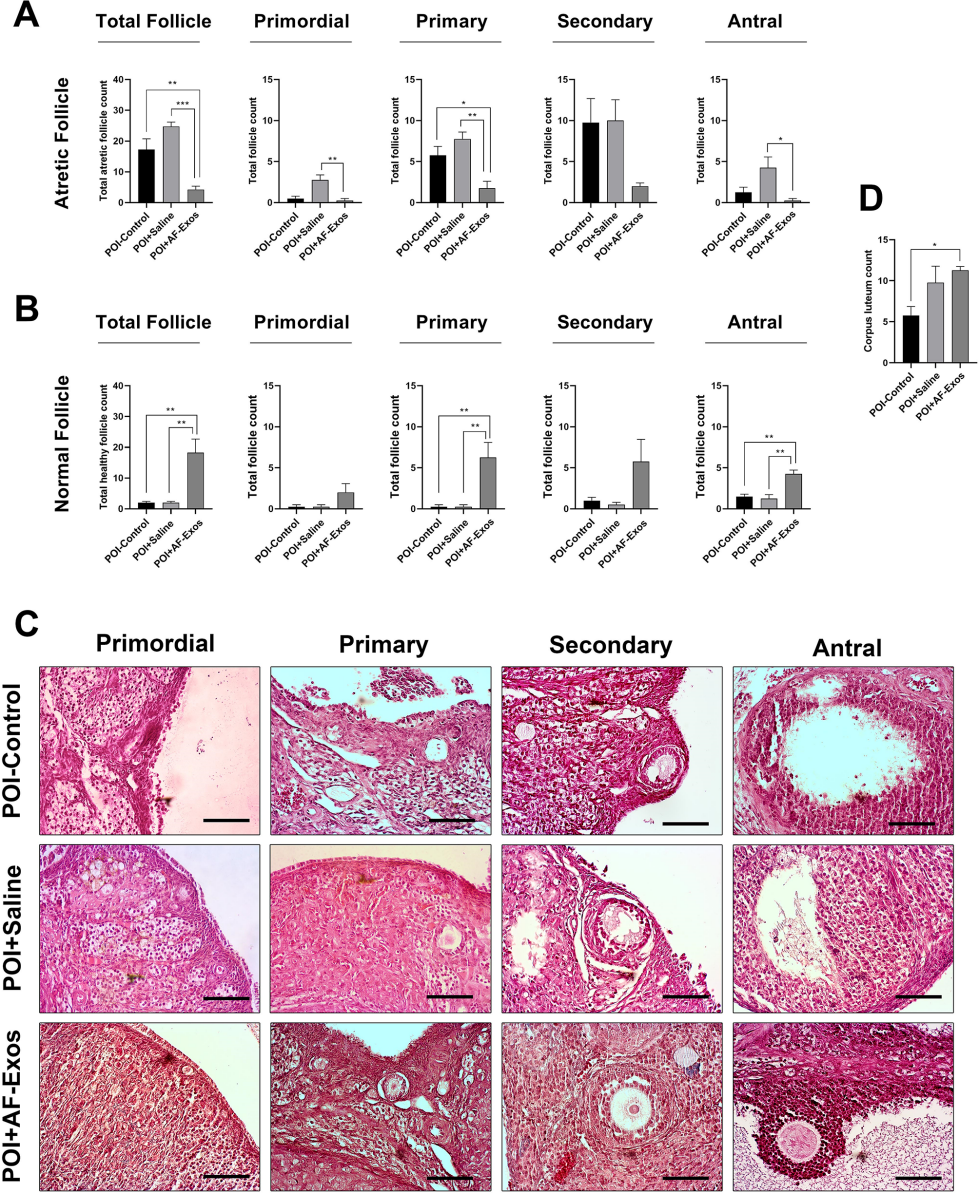
Morphological assessment of ovarian tissue after AF-Exos transplantation, total atretic and atretic follicles count at different developmental stages of primordial, primary, secondary and antral **(A)**, total healthy and healthy follicles count at different developmental stages of primordial, primary, secondary and antral **(B)**, H&E staining **(C)** and total corpus luteum count **(D)**. (Scale bar = 200 µm), *p < 0.05; **p < 0.01 and ***p < 0.001 (n = 4)

To assess the regenerative potential of AF-Exos on ovarian tissue, H&E staining was performed four weeks after AF-Exos transplantation. The data revealed that the total number of atretic follicles significantly decreased after the administration of AF-Exos compared to the control POI group (p < 0.01; Fig. 3A-C). Along with this, the total number of morphologically healthy follicles increased in POI rats after the injection of AF-Exos (p < 0.01; Fig. 3A-C). General improvement was observed regarding the increase in the number of morphologically normal follicles and the decrease in the number of atretic follicles in all stages of growth (primary, secondary, and antral) in the AF-Exo-treated rats compared to the sham group (p < 0.05; Fig. 3A-C). The CL numbers were also significantly increased in POI rats after Exos transplantation compared to the sham group (p < 0.05; Fig. 3D). These data indicate the protective properties of AF-Exos on atretic follicles.

### AF-Exos transplantation and ovarian tissue fibrosis

**Fig. 4:**
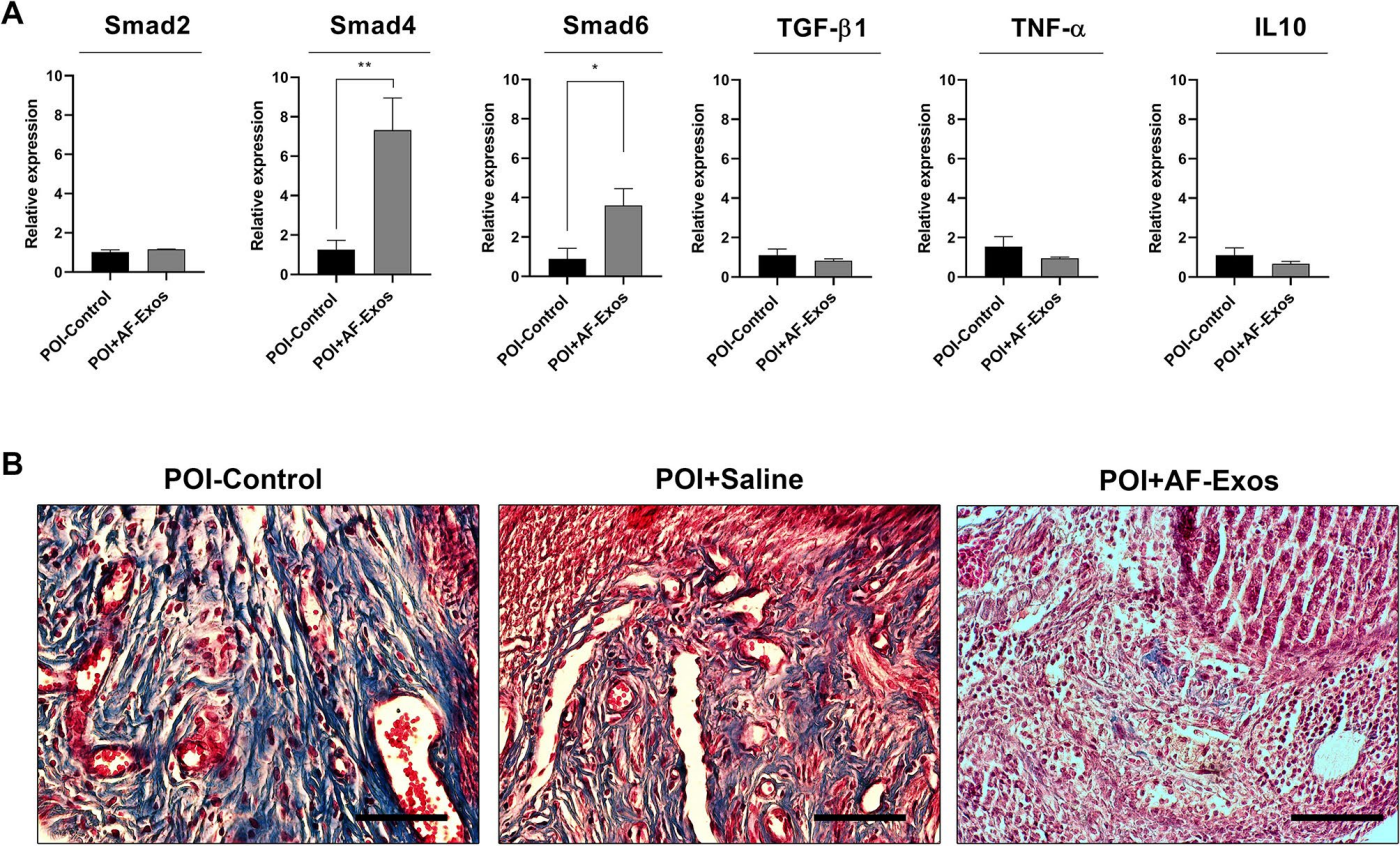
Relative expression of *Smad-2, Smad-4, Smad-6, Tgf-β1*, *Tnf-α*, and *IL-10* genes in ovarian tissue **(A)**. Masson trichrome staining **(B)** after intervention. (Scale bar = 200 µm), *p < 0.05; and **p < 0.01 (n = 4)

The expression of fibrosis-related genes was monitored following AF-Exos administration in POI rats (Fig. 4). The data showed that the expression of Smad-4 and − 6 increased following intervention in the AF-Exos group compared to the control POI rats (p < 0.01 and p < 0.05 respectively; Fig. 4). Additionally, the expression of Tgf-β1, Tnf-α, and IL-10 was reduced after AF-Exos administration, but the differences were not statistically significant. Masson’s trichrome staining indicated the presence of blue-colored collagen fibers within the ovarian tissue parenchyma after CTX injection (Fig. 4). It was observed that AF-Exos administration reduced the fibrotic changes by reducing the number of blue-colored fibers. These data demonstrate that AF-Exos possess the regenerative potential to restore the function of POI ovaries by reducing fibrotic changes.

### AF-Exos transplantation modulated the hormone levels in POI rats

**Fig. 5:**
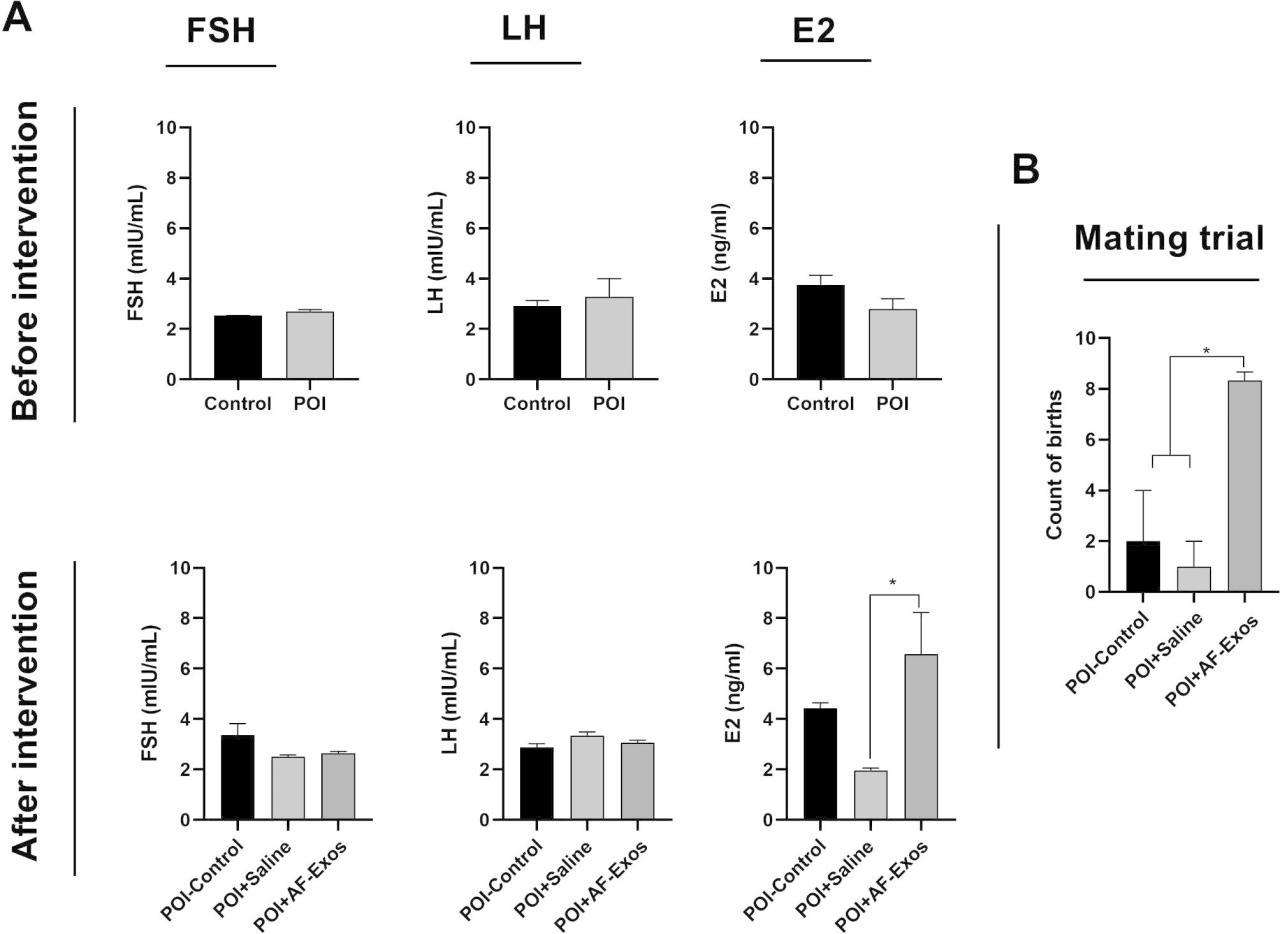
Serum levels of follicle-stimulating hormone (FSH), luteinizing hormone (LH) and estradiol (E2), before also after AF-Exos transplantation, (n = 4) **(A)**, mating trial and litter counts after intervention **(B)**. *p < 0.05 (n = 3)

The serum levels of FSH, LH, and E2 hormones were measured before and after intervention (Fig. 5A). An elevated pattern was noticed in the systemic levels of FSH and LH hormones in the POI rats compared to the control group, but the differences were not statistically significant (p > 0.05; Fig. 5A). Upon AF-Exos transplantation, the serum levels of FSH and LH hormones decreased; however, the differences were again not significant (p > 0.05; Fig. 5A). Concerning E2 levels, it was indicated that the injection of AF-Exos led to an increase in E2 levels compared to the control POI and POI + Normal Saline group (p < 0.05; Fig. 5A). These data showed that AF-Exos can restore the basal levels of certain sex-related hormones in POI rats after four weeks.

#### AF-Exos transplantation improved fertility status

Four weeks after AF-Exos treatment, three remaining rats from each group were mated with fertility-proven male rats. After the gestation period, the total number of births was recorded. Interestingly, all three rats in the AF-Exos group gave birth to 8, 9, and 8 litters, respectively. In the sham and POI-control groups, only one rat gave birth to 3 and one to 6 litters, respectively (p < 0.05; Fig. 5B). These data indicate that AF-Exos transplantation improved fertility status in POI rats.

## Discussion

In dealing with POI patients, clinicians, and reproductive biotechnologists have been focusing on developing cell-free therapeutics with minimal side effects, due to the efficiency and safety concerns of available therapeutic modalities. However, few studies have analyzed the effects of Exos on the treatment of POI conditions [49]. In our experiment, we used AF as the source of Exos. It is well established that amniotic fluid is a rich source of stem cells and contains numerous factors with regenerative outcomes [50]. We assessed the regenerative potential of AF-Exos in terms of POI ovarian function in a rat model.

The data show that injection of CTX with the current protocol can induce POI-like conditions in rats that can be applied to human counterparts [47, 49, 51]. CTX is an alkylating agent with the potential to affect both resting and dividing cells in female reproductive organs, especially ovaries [11]. Consistent with this claim, histological examination revealed the reduction of healthy follicles and the progression of atretic changes in rats, leading to the development of POI conditions. Likewise, the levels of sex-related hormones such as FSH, LH, and E2 remained unchanged after the induction of POI. One reason for this could be that the current protocol only induced a non-overt POI model using the CTX injection, and the reduction of FSH, LH, and E2 levels can be compensated with the activity of the hypothalamic-pituitary-ovarian axis in response to the pathologies that occurred inside the ovaries [52, 53]. The levels of sex-related hormones can vary in POI models depending on the primordial follicle pool and rapid follicle exhaustion [52]. Despite the lack of significant differences in systemic levels of FSH, LH, and E2 in POI rats, Masson’s trichrome and H&E staining revealed progressive fibrotic changes and atretic follicles in all developmental stages.

The injection of AF-Exos prevented follicular atresia induced by CTX. Along with these changes, the number of morphologically healthy follicles increased compared to the control POI group. Consistent with the current data, Huang and colleagues reported that human adipose tissue mesenchymal stem cells (MSCs) Exos can increase the number of follicles to almost normal levels in four stages (primordial, primary, secondary, and antral) with the normalization of sex-related hormones [49]. It was suggested that Exos can reduce apoptotic changes via the inhibition of Fas, FasL, Caspase-8, and Caspase-3 [49]. Our data indicated the non-significant reduction of TNF-α and IL-10, which can lead to the reduction of follicular atresia. It is also possible that the protective effects of injected Exos are associated with the restoration of granulosa cell function under pathological conditions. Based on previous data, MSCs can increase the proliferation of granulosa cells in a paracrine manner in vitro conditions [49]. Likewise, Exos from other stem cell sources, such as human umbilical cord MSCs, can promote the activity and proliferation of granulosa cells via the regulation of the Hippo pathway, as indicated by ErdU and CCK-8 assays [54]. Zhang and co-workers found the induction of DAZL and FOXL2 in POI rat ovaries with the reduction of apoptosis that received menstrual blood-derived stem cell Exos [55]. The induction of the mTOR-dependent signaling pathway is another therapeutic effect associated with the administration of Exos. Yang and colleagues have indicated that the suitable uptake of human umbilical cord MSCs via primordial follicles further activates the PI3K/mTOR signaling pathway, which is crucial for the therapeutic effects of Exos [56]. The modulation of several signaling pathways in POI ovaries indicates the pleiotropic properties of Exos in the restoration of ovarian tissue function. Another interesting finding in this study is the reduction of fibrotic changes. Cen et al. have reported that after femustine treatment in POF model rats, the expression of SMAD2 and GDF-9 genes was increased [57]. In line with current data, Huang and co-workers have indicated a reduction in fibrotic changes in POI rats after the injection of adipose tissue MSC Exos, where in vitro and in vivo analyses have revealed reduced expression of SMAD2, SMAD3, and SMAD5 [49].

In this study, we have found a statistically significant up-regulation of the TGF-β signaling pathway inhibitory effector SMAD-6, while concurrently the expression of SMAD-4 has also increased. Despite the reduced expression of TGF-β and IL-10, these values did not reach statistically significant levels. One possible reason for the induction of SMAD-4 is that this factor acts as a stimulatory effector in TGF-β signaling pathway factors and concurrently can regulate lipogenesis activity. To be specific, studies have shown that SMAD-4 is a key regulator in androgen and estrogen production and FSH production via estrogen production [58, 59]. Regarding the data from Masson’s trichrome staining and real-time PCR analysis, we can hypothesize that AF-Exos can attenuate the fibrotic changes within the ovarian tissue with POI. It is also suggested that the increase in the stimulatory TGF-β signaling factor SMAD-4 is related to other biological effects of this factor. More investigations are mandatory to precisely address the pleiotropic effects of SMAD-4 under fibrotic changes. The lack of significant data in terms of TNF-α and IL-10 could be due to the short duration of the experiment, while in various studies, inflammatory factors were reduced after treatment in the POF model [47, 51, 60].

We have also noted an increase in E2 in POI ovaries after the injection of AF-Exos. In line with our data, Yang and co-workers have indicated improved ovarian function after transplantation of bone marrow MSC Exos in a rat model of POF [61]. This study reported normal levels of FSH, LH, E2, and AMH hormones after transplantation. Finally, the fertility of POI rats treated with AF-Exos was checked after one month. The data showed an enhanced fertility rate in POI rats that received AF-Exos compared to control-matched groups. In support of our data, previous studies have also reported that Exos can heal damaged follicles and increase the fertility rate [61–65].

The current experiment faces several limitations that need consideration for future investigations. Despite a sufficient amount of AF-Exos, it should be noted that the heterogeneity in isolated Exos is high in AF samples compared to the other cultured cell samples in laboratory settings. Enrichment of Exos should be carefully monitored in terms of insidious infections and genetic aberrancies before application in in vitro and in vivo conditions. Besides, the mucoid appearance and varied floating particles in AF make the Exo isolation more laborious compared to the other biofluids.

## Conclusions

In conclusion, the administration of Exos derived from stem cells has shown promising therapeutic effects in the treatment of premature ovarian insufficiency (POI). Exos have been found to improve ovarian tissue function by modulating several signaling pathways, including the mTOR-dependent signaling pathway and the PI3K/mTOR signaling pathway. They have also been found to reduce fibrotic changes within the ovarian tissue, as evidenced by reduced expression of SMAD2, SMAD3, and SMAD5 genes.

In addition, Exos have been found to increase the expression of SMAD-6, an inhibitory effector of the TGF-β signaling pathway, which may contribute to the attenuation of fibrotic changes within the ovarian tissue. This effect may be related to other biological effects of SMAD-4, a stimulatory effector of TGF-β signaling, which has been found to play a key role in androgen and estrogen production, FSH production, and lipogenesis activity. These data indicate that Exos harbor distinct signaling cytokines and growth factors with the potential to promote the reparative mechanism in POI rats via the regulation of fibrotic changes and chronic inflammation, indicating the close relationship between the molecular biology and female fertilization rate [66, 67]. Whether and how the metabolic status of parent cells can affect the regenerative potential of isolated Exos in terms of fertility should be addressed in future studies.

Furthermore, Exos have been found to increase the levels of estrogen in POI ovaries and enhance fertility rates in POI rats. These findings suggest that Exos may be a promising therapeutic strategy for the treatment of POI, and further studies are warranted to fully elucidate their mechanisms of action and potential clinical applications. Overall, these findings have important implications for the development of novel treatments for POI and may offer hope to women suffering from this condition.

## Data Availability

Data are contained within the article. Datasets related to this project can be obtained from the corresponding author based on a reasonable request.
