# Correction: Amniotic fluid-derived exosomes attenuated fibrotic changes in POI rats through modulation of the TGF-β/Smads signaling pathway

**DOI:** 10.1186/s13048-024-01488-z

**Published:** 2024-08-19

**Authors:** Nahideh Nazdikbin Yamchi, Shahin Ahmadian, Halimeh Mobarak, Farhad Amjadi, Rahim Beheshti, Amin Tamadon, Reza Rahbarghazi, Mahdi Mahdipour

**Affiliations:** 1 Stem Cell Research Center, Tabriz University of Medical Sciences, Tabriz, Iran; 2 Faculty of Veterinary Medicine, Shabestar Islamic Azad University, Shabestar, Iran; 3 PerciaVista R&D Co., Shiraz, Iran; 4 Department for Scientific Work, Marat Ospanov Medical University, West, Aktobe, Kazakhstan; 5 Drug Applied Research Center, Tabriz University of Medical Sciences, Tabriz, Iran; 6 Department of Applied Cell Sciences, Faculty of Advanced Medical Sciences, Tabriz University of Medical Sciences, Tabriz, Iran; 7 Department of Reproductive Biology, Faculty of Advanced Medical Sciences, Tabriz University of Medical Sciences, Tabriz, Iran

Correction: J Ovarian Res 16, 118 (2023)

10.1186/s13048-023-01214-1

Following publication of the original article [1], the authors reported that Figs. 3 and 4 have been updated due to unintentional errors when compiling the data. The correct figures were included in this correction.

**Corrected figure 3:**

**Figure:**
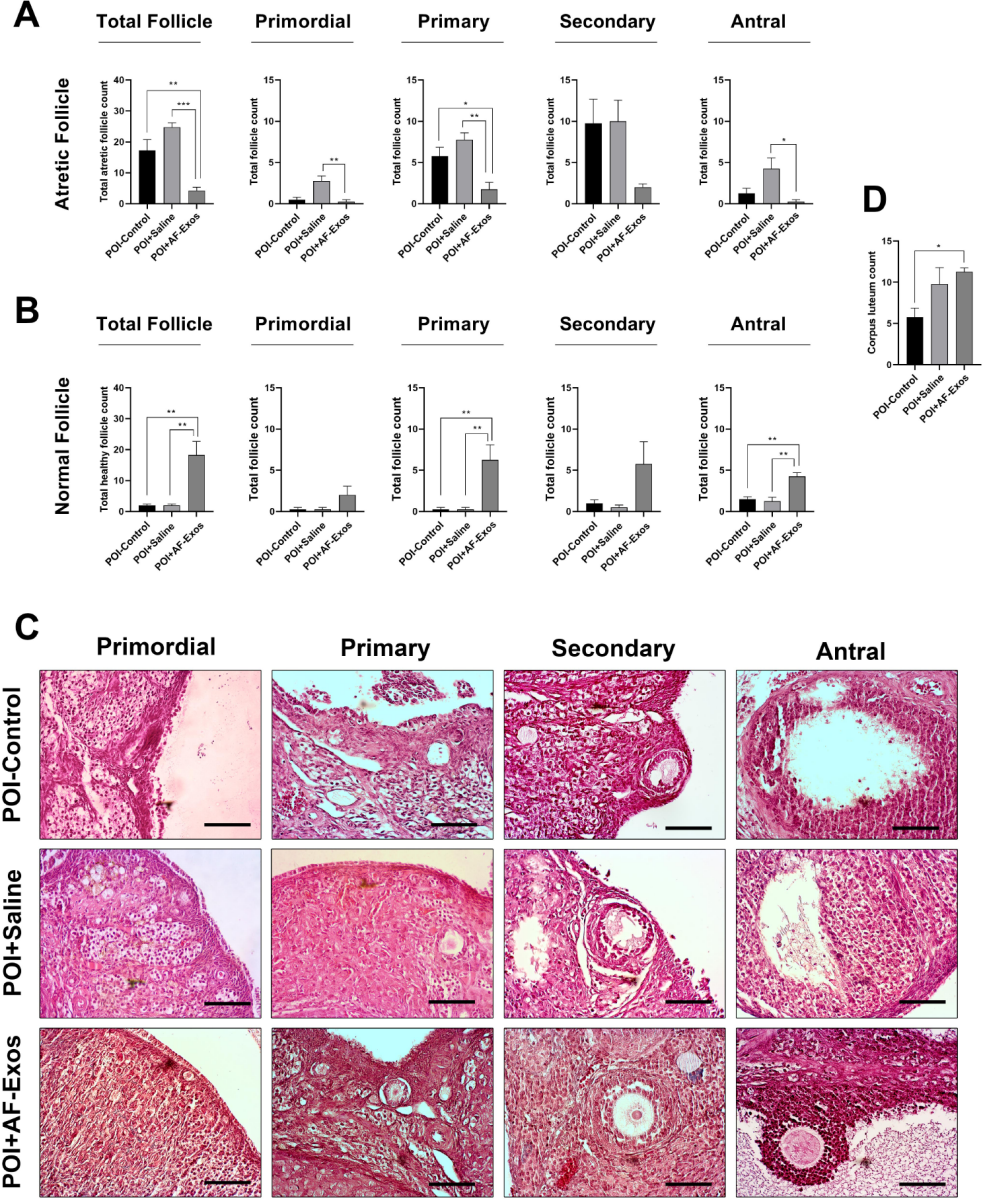

**Corrected figure 4:**

**Figure:**
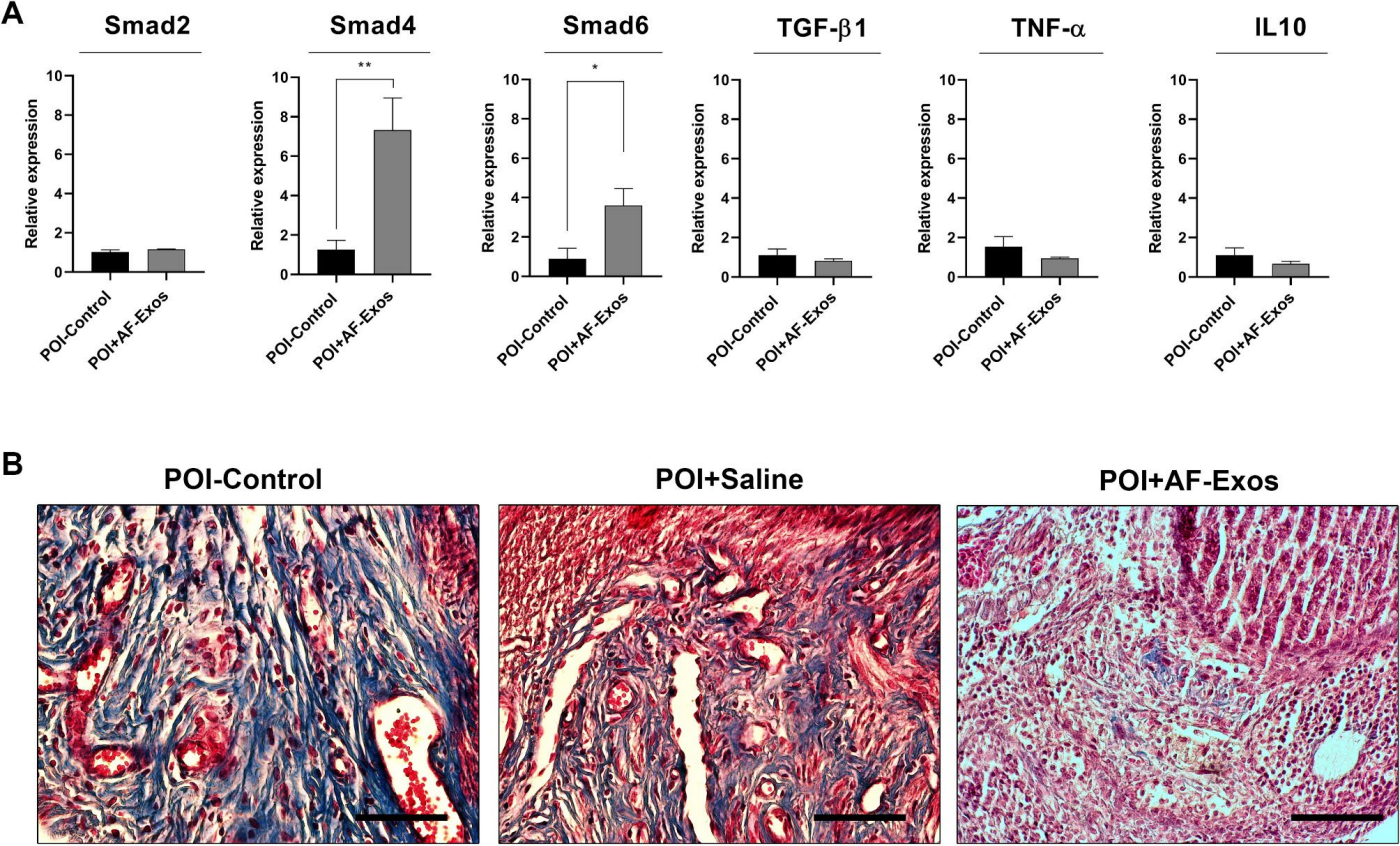

The original article [1] has been corrected.
